# Are Crohn’s Disease Patients Limited in Sport Practise? An UltraEndurance Case–Control Study Response

**DOI:** 10.3390/ijerph191610007

**Published:** 2022-08-13

**Authors:** José Francisco Tornero-Aguilera, Joaquín Sánchez-Molina, Jose A. Parraca, Ana Morais, Vicente Javier Clemente-Suárez

**Affiliations:** 1 Faculty of Sports Sciences, Universidad Europea de Madrid, 28670 Madrid, Spain; 2 Research Center in Applied Combat (CESCA), 45007 Toledo, Spain; 3 Departamento de Desporto e Saúde, Escola de Saúde e Desenvolvimento Humano, Universidade de Évora, 7004-516 Évora, Portugal; 4 Comprehensive Health Research Centre (CHRC), University of Évora, 7004-516 Évora, Portugal; 5 Grupo de Investigación en Cultura, Educación y Sociedad, Universidad de la Costa, Barranquilla 080002, Colombia

**Keywords:** sport, exercise, disease, HRV, autonomic modulation

## Abstract

The aim of this study was to analyze the psychophysiological response of a Crohn’s Disease patient in an ultra-endurance event. The psychophysiological responses of a Crohn’s Disease and non-Crohn’s Disease participant were analysed before during and after an 8 h ultra-endurance running event. Results showed how Crohn’s patient presented a similar psychophysiological response than non-Crohn’s participant in the ultra-endurance event, except for a higher pre- and post-event sympathetic modulation, lower event sympathetic tone, and lower event body temperature. This study could contribute to improving physical activity recommendations for persons with Crohn’s Disease and open a new research line for an improved understanding of psychophysiological modifications of Crohn’s Disease patients during exercise.

## 1. Introduction

Crohn’s disease is a chronic relapsing inflammatory disorder mainly affecting the gastrointestinal tract with extraintestinal manifestations and is associated with immune disorders, leading to fatigue, unfitness, or a decreased quality of life [1]. With an overall incidence of 2.5–3 million people in Europe, healthcare costs are estimated at EUR 4.6–5.6 billion per year [2]. Despite the suggested benefits of exercise training in the prevention and management of chronic diseases and its associated medical-cost saving [3], including cardiovascular disease, diabetes mellitus, and rheumatoid arthritis [4], guidelines for exercise prescription for patients with Crohn’s disease are scacre. However, recent studies recommended physical exercise as an adjunct therapy for Crohn’s Disease patients [5,6,7]. Empirical evidence on the effects of exercise training in Crohn’s Disease is sparse, and most of the actual literature focused on either low-moderate intensity [8,9,10] or high-intensity training [11].

In this line, the positive effect that exercise has on chronic conditions, such as Crohn’s Disease, derives from its potential for improving patients’ physical health condition and quality of life. Health improvements allow an increased immune response, leading to greater tolerance and increasing stress-management and motivation [12,13]. There are some major challenges in simply maintaining fitness levels and taking new collective classes or performing fitness activities when experiencing symptoms of uncontrollable diarrhea, fatigue, joint problems and severe pain are at their worst, and these are challenging to normally healthy people and appear insurmountable to those with Crohn’s Disease [14]. Thus, motivation can be a key factor when it comes to tolerance and resilience to the acute symptoms caused by Crohn’s Disease.

Ultra-endurance races have a highly motivating and attractive unique element, and this is a fact reflected in the exponential growth of its participation over the last decade in the general population [15]. However, a great incidence of gastrointestinal issues in these events has been reported and raised concerns regarding the impact of prolonged strenuous exercise on gastrointestinal health [16]. Therefore, there may be greater concerns for the progression or development of complications such as septic shock, colitis, paralytic ileus, ischemic bowel, and worsening of Crohn’s Disease. Explanations may be related to either the circulatory-gastrointestinal pathway involving the redistribution of blood flow to working muscles and peripheral circulation, which aids skeletal muscle metabolism and thermoregulation, subsequently reducing total splanchnic perfusion. This also can include neuroendocrine-gastrointestinal pathways involving an increase in sympathetic activation, reducing overall gastrointestinal functional capacities [16].

Ultra-endurance events are becoming a new personal challenge, in which many patients and people with limitations prove that the capacity for sacrifice and efforts can reach very high limits to themselves and to those who suffer the same pathology. In this sense, even Paralympic athletes have faced the challenges of ultra-endurance challenges, showing how, even with limitations, willpower and motivation are fundamental for achieving these challenges [17]. To the best of our knowledge, there are no studies on Crohn’s Disease in ultra-endurance events. Thus, we conducted the present research with the aim to analyze the psychophysiological response of a Crohn’s disease patient in an ultra-endurance event.

## 2. Case Report

Two volunteer male participants were analysed: one with Crohn’s Disease and another one without it. The characteristics of the participants are presented in Table 1. Athletes participated voluntarily and signed an informed consent form following the procedures of the Helsinki Protocol. The study was approved by the University Bioethics Committee (CIPI/002/17).

## 3. Methods

### 3.1. Ultra-Endurance Event

**Figure 1:**
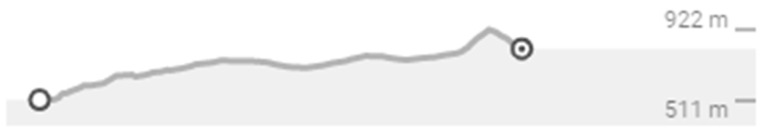
Altimetry profile of the ultra-endurance event.

The event lasted 44 km with an accumulated positive altitude change of 503 m and an accumulated negative altitude change of 208 m (see Figure 1). The duration of the event was 8 h and 8 min.

### 3.2. Evaluation of the Participants

**Figure 2:**
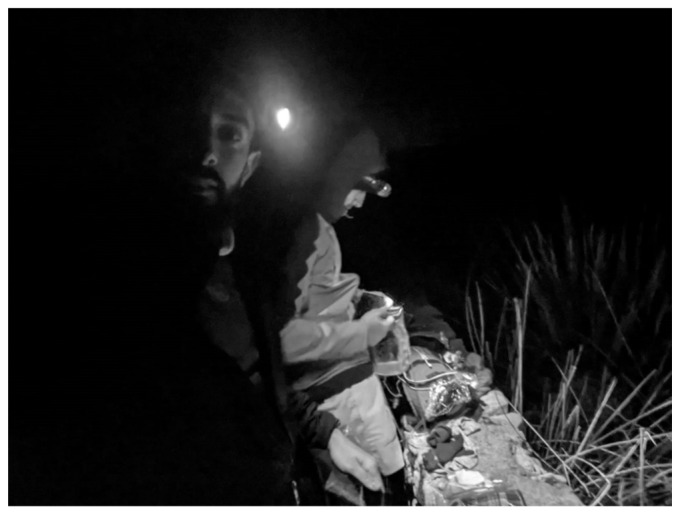
Data collection during the ultra-endurance event.

There were three evaluation moments:

1. Thirty-two hours prior to the ultra-endurance event, analysing basal heart rate variability (HRV). Data were collected with the participants lying in a bed after waking up (8:00 a.m.);
2. Seven hours prior to the ultra-endurance event, analysing HRV and body composition parameters. Data were collected with participant lying in a bed after waking up (8:00 a.m.);
3. Ultra-endurance event: At 14:45 p.m. 1 h pre-event, HRV was collected (20 °C, 20% humidity). From 15:45 to 23:54 p.m., race HRV was collected (see Figure 2) (13 °C, 82% humidity), and from 23:54 p.m. to 00:54 a.m., post-event HRV was collected (5 °C, 85% humidity). Before, during, and after the ultra-endurance event, the following parameters were analysed: rating of perceived exertion, leg pain, blood glucose, blood oxygen saturation, heart rate, temperature, urine colourimetry, urine nitrates, protein, glucose, and pH. Hand strength, leg strength, and respiratory muscle strength were analysed before and after the event. All analyses were conducted following previous procedures and analysis systems [18].

### 3.3. Study Variables

We analysed the following variables:

- Rating of perceived exertion (RPE), Borg 6–20 scale [19];
- Perceived leg muscle pain in a self-reported 1–100 scale [17];
- Blood glucose concentration by the analysis of 5 µL of capillary finger blood using a portable analyser (One Touch Basic, LifeScan Inc., Madrid, Spain);
- Body temperature (BT) was measured by an armpit thermometer (digiT-40, Cammi Group SpA. Calvisano, Italia);
- Blood oxygen saturation and HR by a pulse oximeter (PO 30 Beurer Medical);
- Respiratory muscle strength by the forced vital capacity (FVC) using a QM-SP100 (Quirumed, Spain) spirometer in a maximum inhale–exhale cycle;
- Isometric hand strength by a grip dynamometer (Takei Kiki. Koyo, Japan);
- Leg strength manifestation was analysed by a horizontal jump test. Subjects performed a standardized warm-up comprising 2 × 10 vertical jumps with 30 s of recovery, and then, they performed two maximal horizontal jumps using the best attempt for the statistical analysis [20];
- Urine samples were collected to analyse dehydration levels by the urine colour chart (colour range 1–8; where 1 = very pale-yellow urine, reflected a good level of hydration, and 8 = very dark yellowish brown, reflected a significant level of dehydration) [21]. Urine nitrates, protein, glucose, and pH were measured with the Urine Combur-Test (Roche, Madrid, Spain) stripes [22];
- Body mass index (BMI), muscle mass, and fat mass were determined by using bioelectrical impedance analysis (InBody 720, Biospace Co., Ltd., Seoul, South Korea) [23];
- HRV measurements were recorded by a validated (Giles, Draper, and Neil, 2015) Polar V800 heart rate monitor (Polar, Kempele, Finland). The R-R series was analysed using Kubios HRV^®^ software (version 2.1, Biosignal Analysis and Medical Imaging Group, University of Kuopio, Kuopio, Finland). The following variables were analysed: minimum, average, and maximal hear rate (HR, bpm); square root of the mean of the sum of the squared differences between adjacent normal R-R intervals (RMSSD, ms); percentage of differences between adjacent normal R-R intervals more than 50 milliseconds (PNN50, count); low (HF) and high (HF) frequency bands (LF) in normalized units (nu); LF/HF ratio; sensitivity of the short-term (SD1, ms) and long-term (SD2, ms) variability of the non-linear spectre of the HRV, and the approximate entropy of HRV (ApEn, ms).

## 4. Results

Results for both participants are presented in Table 2 and Table 3. We found a similar response between participants in the variables analysed except for a higher pre- and post-event sympathetic modulation, lower event sympathetic tone, and lower event body temperature for the Crohn’s Disease participant.

Participants have ab libitum access to drinking and eating during the ultra-endurance event. The Crohn’s Disease participant drank 1.6 L of water and an ingestion of 580 kcal, 22 g of protein, 31 g of carbohydrates, and 14.1 g of fat. The non-Crohn’s Disease participant drank 1.8 L of water and presented an ingestion of 27 g of protein, 52 g of carbohidrates, and 16.1 g of fat.

## 5. Discussion

Few studies have approached the influence of ultra-endurance events on population suffering from chronic conditions. To the best of our knowledge, this is the first study to report the psychophysiological response of a Crohn’s Disease patient in an ultra-endurance event.

Blood and urine markers have been explored as indicators of intestinal inflammation in Crohn’s disease. In our case report, urine markers (colorimetry, nitrates, pH, protein, and urine glucose) remained stable during the 8 h event duration in both study subjects. Another urological complication in patients with Crohn’s disease is the formation of fistulas or communications between the digestive tract and the urinary system (uterovesical fistulas) [24]. The most common signs and symptoms include discomfort located right above the pubis, dysuria (pain when urinating), and urinary infections; in some cases, air or feces from the intestine may appear in the urine [24,25]. In our case study, the subject did not reveal any symptoms. Therefore, despite the effort required and muscle exhaustion, the activity carried out did not trigger symptoms of this clinical complication. In addition, as expected and observed in the results, as blood glucose levels decreased, urine levels slightly increased. Some of these data differ from those previously observed in the literature. Thereby, the incidence of exercise-associated hyponatremia in ultra-endurance races was higher than thought and was not even counteracted with prior-to-race over-hydration [26].

Strength manifestation by contrast to previous markers suffered a great variation. Despite the poor understanding of fatigue, it (and a reduction in aerobic capacity) is a frequently reported symptom in Crohn’s disease in addition to a decrease in muscle strength [5,27]. Both hand grip and lower limb strength manifestations decreased significatively at the end of the event in both study subjects. These values are in line with the increases in the rate of perceived exertion and leg-pain perception. These parameters are crucial for revealing the intensity of the activity and how, despite it, several of the Crohn’s disease clinical markers remained regular, thus showing real possibilities for the Crohn’s disease population to confront ultra-endurance trials. However, more in-depth and longer investigations are needed with respect to studying the influence of these events in aspects such as bone mineral loss, intestinal problems, body composition, sleep disturbances, or anemia.

The ultra-endurance event produced a large perception of exertion and leg pain in both subjects analyzed, showing the large demand of the event. These results were in line with previous studies in other ultra-endurance events [28]. In this line of research, the decreases in hand, leg, and expiratory muscle-strength manifestations and the decrease in flexibility were similar in both subjects and in line with previous reports in ultra-endurance events [29]. These modifications were previously related with large muscle breakdowns and protein catabolism, which only slightly increased urine protein and glucose presence in Crohn’s Disease participants but was maintained within a range that was previously reported in ultra-endurance events [30].

Glucose metabolism disturbances have been reported to some extent among individuals with Crohn’s Disease in addition to the release of gut hormones enrolled in glucose homeostasis [31]. The complex nature and etiology of this chronic disease renders the most appropriate therapy uncertain, but frequent treatments include corticosteroids, which may cause high levels of blood glucose [32]. However, by the time the first glucose measure is performed, which is normally after the subjects´ lunch, the levels were lower than 10 mmol/L. These are clinical values recommended by the American Diabetes Association (ADA) [33] and expected for a non-diabetic individual after a meal. As the race developed, glucose values ranged on average between 4.4 and 4.8 without differences between both subjects, thus showing very even glycolic metabolism and without clinical alterations. Taking into account that Crohn’s patients experience alterations in the glycolytic profile and homeostasis [31], this result could be unexpected, and the effects of chronic exercise and endurance training may explain it [34].

Skin temperature presents a wide spectrum of values due to interpersonal differences in thermoregulation as well as its behavior during physical activity. The main thermoregulatory role of thermal cutaneous signals is to provide auxiliary feedback to the thermoregulation system, reducing the system’s response time and making the body’s temperature stable [35]. The Crohn’s disease subject’s temperature presented a slight decrease. This temperature behavior may be explained due to the exacerbated stress response presented by the Crohn’s disease subject, which is reflected via the sympathetic activation shown in HRV analysis. In this line, while there is a prolonged effort, acute stress triggers peripheral vasoconstriction, causing a rapid, short-term drop in skin temperature homeotherms [36]. This response was previously observed in highly physically demanding and stressful events [37]. However, in ultra-endurance [29] events, the opposite skin response was presented amongst the extensive literature [17,29,38,39]. The decrease in hydration status and the increase in metabolic energy output caused this increase in body temperature [40]. The lack of perceived stress and its somatization by the non-Crohn’s disease subject produces a correspondence between skin temperature behavior and those found in the previous literature [17].

Regarding the interaction between the autonomous nervous system and the cardiovascular system, it is known that long-term training increases HRV [41] (i.e., boosting the increase in parasympathetic tones while ensuring sympathetic tone inhibition in the resting state). On the other hand, the decrease in HRV is used as a marker of overtraining, exhaustion, or even disease progression [42,43]. The individual analysis shows how the Crohn’s disease patient presented significantly higher basal LF prior to the event. These values report a sympathetic nervous system activation, and it is related with overtraining, muscular injuries, and/or health disorders [43,44] or an anticipatory anxiety response, which are also evaluated in other sport events and before demanding activities such as parachuting, military operations, or aircraft and helicopter flights [45,46]. During the ultra-endurance event, previous sympathetic hyper-activation decreased, maintaining high sympathetic modulations such as the low RMMSD, PNN50, and SD1 shown, but a decrease in anticipatory anxiety responses was also observed. This autonomic response of both the Crohn’s disease patient and the control subject was in line with previous ultra-endurance event observations [17,28,29]. The high demands of the ultra-endurance event are reflected in the high values of sympathetic activations in the hour after completion, showing high values of LF in both the Crohn’s disease patient and the control subject.

Oddly enough, during the trial, the ratio of LF/HF and the relationship between vagal tone and prior anxiety markers became balanced. In this line, there are no markers and values related to disease, overtraining, or exhaustion; thus, subjects with inflammatory diseases who want to attend an ultra-endurance event need to understand and attend carefully to their own sensations of pain and inflammation. The non-Crohn’s disease subject, however, presented great sympathetic activations before the event, but this anticipatory response, far from being diminish or realigned, became more exacerbated and significant during the event; yet, one hour after the end of the event, the subject reflected sympathetic activations, which is result of the difficulty of the test.

Regarding the function and respiratory capacity of the subjects, both the data and the percentage of change were almost identical in the subjects. The forced vital capacity was analyzed since, in certain pathologies, it is possible that the forced air capacity of the lungs may be lower than the vital capacity during slower exhalation [47]. Results evinced how the respiratory capacity of both subjects was significantly affected as a consequence of the ultra-endurance event, but there were many differences between them.

Normally, both athletic and nonathletic population after any unaccustomed or intense exercise will experience pain 24–72 h post-exercise. This form of damage is characterized by muscular pain and discomfort, decreased muscle force production, and a reduced range of motion [48]. The extension and nature of the ultra-endurance test triggered the onset muscle soreness given the fact that these damage signals came out by the end of the trial. The onset of muscle soreness increased the risk of injury and strain placed on soft-tissue structures. A reduced range of motion, as evinced by the end of the event, may lead to a reduced ability to tolerate the load and a reduced ability to adjust to changes brought about by physical activity, such as modifications in motor control [49]. By attending to the subject’s response, we observed a rough change from the pain perceived in the middle of the race with respect to the end. Previous investigations evinced the possible unconsciousness and misperception of one’s own effort when confronting tough situations [28,29,50,51,52,53,54,55,56,57,58]. Thus, a greater and more continuous control of the perception of effort is recommended since these subjects are at certain risks, and as we have shown, their own experience and control will be key to the prevention of injuries and symptoms related to their disease: cramps, inflammation, and sharp pain. However, even though there were significative changes in strength values, lower limb flexibility, and RPE, these changes likewise affected both study subjects, supporting and strengthening the chances of Crohn’s disease patients in participating in sports and even in ultra-endurance events.

Finally, there is a need for further research in exercise and Crohn’s disease, and a form of standardized reporting with respect to exercise programs is needed to enable the evaluation and implementation of research results. If guidelines are to be evidence-based, there is a need for the rapid expansion of research in this area. Despite some theoretical barriers for Crohn’s disease patients among these types of events, our results suggest that our findings are dependable, showing similar psychophysiological responses in both the Crohn’s disease patients and the control subject. However, in order to enable patients in varying exercise levels according to their disease activity, the effects of medication, and the severity of symptoms, there is a need to test individually prescribed exercise for subjects with Crohn’s disease.

### Limitation of the Study

The main limitation of the study was that the analysis only involved one Crohn’s Disease patient. A large sample was precluded due to the difficulty in finding patients willing to participate in ultra-endurance events. An analysis of stress hormones such as cortisol or alpha amylase would help in understanding the psychophysiological stress response of participants, but economical issues limited the analysis of these hormones [59,60,61].

## 6. Conclusions

With the present results, we can conclude that the Crohn’s Disease patient presented a similar psychophysiological response than the non-Crohn’s Disease participant in an ultra-endurance event, with the exception of higher pre- and post-event sympathetic modulations, lower event sympathetic tone, and lower event body temperature. This information can help improve actual Crohn’s Disease physical activity recommendations and open a new direction of research in order to better understand the psychophysiological modifications of Crohn’s Disease patients during exercise.

## Figures and Tables

**Table 1:** Main characteristics of the participants with and without Crohn’s Disease.

	Montreal Crohn’s Disease Classification *	Age (Years)	Height (cm)	Weight (kg)	Fat Mass (kg)	Muscle Mass (kg)	Training (min/Week)
Crohn’s disease participant	A3 L3 B1	41	178	77.7	17.7	33.7	322.4
Non-Crohn’s disease participant	-	36	174	68.8	13.1	31.6	350.3

* A3 (>40 years); L3 (ileocolonic); B1 (non-structuring, non-penetrating).

**Table 2:** Modification of study variables before, during, and after the ultra-endurance event.

	**Time**	**Urine Colourimetry**		**Urine Nitrates**		**Urine pH**		**Urine Protein (mg/dL)**		**Urine Glucose (mg/dL)**	
Crohn’s Disease participant	14:16	2		0		5		0		0	
	19:45	1		0		5		1		1	
	23:56	2		0		5		1		1	
Non-Crohn’s Disease participant	14:16	1		0		5		0		0	
	19:45	1		0		5		0		0	
	23:56	1		0		5		0		0	
	**Time**	**Rating of Perceived Exertion**	**Leg Pain Perception**	**Glucose (mmol/L)**	**Blood Oxygen Saturation (%)**	**Heart Rate (bpm)**	**Temperature (°C)**	**Hand Strength (N)**	**Leg Strength (cm)**	**Forced Vital Capacity (mL)**	**Sit and Reach (cm)**
Crohn’s Disease participant	14:16	7	15	8.2	99	73	35.6	45.5	120	5300	17
	19:45	11	30	4.8	96	69	34.2				
	23:56	20	100	4.6	96	72	34.6	38.1	73	3800	8
Non-Crohn’s Disease participant	14:16	6	5	9.9	96	70	35.9	53.4	182	5200	22.5
	19:45	10	35	4.7	97	63	36.0				
	23:56	20	95	4.5	96	71	37.8	45.2	120	3850	11

**Table 3:** Heart rate variability modifications of participants.

	Evaluation Moment	Mean HR (bpm)	Min HR (bpm)	Max HR (bpm)	RMSSD (ms)	LF/HF Ratio	SD1 (ms)	SD2 (ms)
Crohn’s Disease participant	Pre 32 h	60.7	54.1	78.6	24.3	9.75	17.26	54.0
	Pre 7 h	57.64	51.4	70.3	32.7	9.76	23.1	69.7
	Pre 1 h	82.4	55.5	123.2	52.0	2.41	36.78	66.4
	Event	112	58.2	162.3	23.2	1.8	16.4	28.2
	Post 1 h	78	61.4	105.2	20.5	5.15	14.5	40.8
Non-Crohn’s Disease participant	Pre 32 h	59	51	67	57.6	1.7	40.8	69.5
	Pre 7 h	56	49.4	68.2	36.6	4.46	25.9	63.8
	Pre 1 h	77	49	138	58.7	3.62	41.6	91.3
	Event	113.8	58	166.1	13.1	9.9	9.2	31.2
	Post 1 h	73.5	50	106.8	41.3	17.69	29.3	96.0

(HR); heart rate; (Min HR), minimum heart rate; (Max HR), maximum heart rate; (RMSSD), the square root of the average of the sum of the differences squared between normal adjacent R-R intervals; (LF/HF ratio), the low-frequency/high-frequency ratio; (SD1), sensitivity of the short-term variability; (SD2), sensitivity of the long-term variability.

## Data Availability

All data are presented in the manuscript.
